# Late-Gadolinium Enhancement Interface Area and Electrophysiological Simulations Predict Arrhythmic Events in Patients With Nonischemic Dilated Cardiomyopathy

**DOI:** 10.1016/j.jacep.2020.08.036

**Published:** 2021-02

**Authors:** Gabriel Balaban, Brian P. Halliday, Bradley Porter, Wenjia Bai, Ståle Nygåard, Ruth Owen, Suzan Hatipoglu, Nuno Dias Ferreira, Cemil Izgi, Upasana Tayal, Ben Corden, James Ware, Dudley J. Pennell, Daniel Rueckert, Gernot Plank, Christopher A. Rinaldi, Sanjay K. Prasad, Martin J. Bishop

**Affiliations:** a Department of Biomedical Engineering, School of Biomedical & Imaging Sciences, King’s College London, United Kingdom; b Cardiovascular Magnetic Resonance Unit, Royal Brompton Hospital, London, United Kingdom; c National Heart and Lung Institute, Imperial College London, London, United Kingdom; d Department of Cardiology, St Thomas’ Hospital, London, United Kingdom; e Department of Computer Science, Imperial College London, United Kingdom; f London School of Hygiene and Tropical Medicine, London, United Kingdom; g Institute of Biophysics, Medical University of Graz, Graz, Austria; h Department of Informatics, University of Oslo, Oslo, Norway

**Keywords:** arrhythmic risk, computational modeling, dilated cardiomyopathy, fibrosis, late gadolinium enhancement, CI, confidence interval, CMR, cardiac magnetic resonance, HR, hazard ratio, ICD, implanted cardioverter defibrillator, ICM, ischemic cardiomyopathy, LGE, late gadolinium enhancement, LVEF, left-ventricular ejection fraction, NICM, nonischemic cardiomyopathy, NIDCM, nonischemic dilated cardiomyopathy, SCD, sudden cardiac death

## Abstract

**Objectives:**

This study sought to investigate whether shape-based late gadolinium enhancement (LGE) metrics and simulations of re-entrant electrical activity are associated with arrhythmic events in patients with nonischemic dilated cardiomyopathy (NIDCM).

**Background:**

The presence of LGE predicts life-threatening ventricular arrhythmias in NIDCM; however, risk stratification remains imprecise. LGE shape and simulations of electrical activity may be able to provide additional prognostic information.

**Methods:**

Cardiac magnetic resonance (CMR)-LGE shape metrics were computed for a cohort of 156 patients with NIDCM and visible LGE and tested retrospectively for an association with an arrhythmic composite endpoint of sudden cardiac death and ventricular tachycardia. Computational models were created from images and used in conjunction with simulated stimulation protocols to assess the potential for re-entry induction in each patient’s scar morphology. A mechanistic analysis of the simulations was carried out to explain the associations.

**Results:**

During a median follow-up of 1,611 (interquartile range: 881 to 2,341) days, 16 patients (10.3%) met the primary endpoint. In an inverse probability weighted Cox regression, the LGE–myocardial interface area (hazard ratio [HR]: 1.75; 95% confidence interval [CI]: 1.24 to 2.47; p = 0.001), number of simulated re-entries (HR: 1.40; 95% CI: 1.23 to 1.59; p < 0.01) and LGE volume (HR: 1.44; 95% CI: 1.07 to 1.94; p = 0.02) were associated with arrhythmic events. Computational modeling revealed repolarization heterogeneity and rate-dependent block of electrical wavefronts at the LGE–myocardial interface as putative arrhythmogenic mechanisms directly related to the LGE interface area.

**Conclusions:**

The area of interface between scar and surviving myocardium, as well as simulated re-entrant activity, are associated with an elevated risk of major arrhythmic events in patients with NIDCM and LGE and represent novel risk predictors.

Nonischemic dilated cardiomyopathy (NIDCM) is one of the most common causes of sudden cardiac death (SCD) (1) and is associated with a poor prognosis (2). Implantable cardioverter defibrillators (ICDs) are commonly used to prevent SCD and reduce mortality in patients with NIDCM. However, the selection of patients for ICD therapy remains a significant clinical challenge. This was highlighted by the results of the recent DANISH (Defibrillator Implantation in Patients With Nonischemic Systolic Heart Failure trial (3), which failed to show a reduction in all-cause mortality for ICD treatment in nonischemic patients with a depressed left-ventricular ejection fraction (LVEF), as is recommend by the current clinical guidelines. There is therefore a need to improve risk stratification of patients with NIDCM to improve patient outcomes and reduce unnecessary ICD placements (4).

Contrast-enhanced cardiac magnetic resonance (CMR) allows for the visualization of areas of replacement fibrosis, revealed by late gadolinium enhancement (LGE), which are thought to disrupt the electrical activity of the heart and contribute to SCD. LGE has been detected in approximately one-third of NIDCM cases and has been shown to predict future SCD independently of LVEF (2). Nevertheless, not all patients with LGE will experience arrhythmias, and there has been an effort to risk stratify patients with LGE more precisely. Previous studies have focused on LGE location (5), image entropy (6), and electrocardiogram QRS width (7).

The shapes and sizes of LGE patterns hold promise for risk stratification in nonischemic disease. In particular, Zorzi et al. (8) noted a dangerous “stria” pattern of scarring in athletes, appearing as elongated bands of contiguous LGE. Furthermore, we observed a characteristic dangerous LGE shape in a mechanistic simulation study (9), which we quantified in a set of shape metrics. In particular, the myocardial interface area, defined as the border between LGE and healthy tissue internal to the myocardium, was longer in the LGE patterns for which we could simulate electrical re-entries. We therefore hypothesized that interface area and other LGE shape metrics may be associated with future SCD in patients with NIDCM and LGE. We also sought to use our previously developed in silico investigations to provide plausible mechanistic explanations underlying these associations.

## Methods

### Study population

Patients were referred to the Royal Brompton Hospital for CMR for the evaluation of NIDCM between 2006 and 2015. All patients gave written informed consent, and the study was approved by the National Research Ethics Committee and performed in accordance with the Declaration of Helsinki. Eligible patients had diagnoses of DCM confirmed by an independent consultant cardiologist, based on clinical details and CMR findings. All patients had increased left-ventricular end-diastolic volume indexed to body surface area and reduced LVEF compared with published reference ranges. Exclusion criteria have been comprehensively described elsewhere (5) and in the Supplemental Appendix. Only patients with clear and reproducible areas of LGE were included: specifically, those who had evidence of mid-wall or subepicardial LGE.

### Image acquisition and segmentation

LGE-CMR short-axis images were acquired using a previously described protocol (5). Acquired image resolution was 1.3 to 2.2 mm in plane with 7- to 10.5-mm slice thickness. Images were included if they were located below the outflow tract and had LGE. Areas of LGE were identified by 2 independent expert readers, blinded to clinical outcomes. LGE was considered present if intramural or subepicardial and visible in both phase-encoding directions and in 2 orthogonal views. The borders of the myocardium were delineated in each short-axis slice with LGE. The enhanced areas were then segmented by expert readers using the full-width half maximum (FWHM) technique and semiautomated software (CVI42, Circle Cardiovascular Imaging Inc., Calgary, Alberta, Canada).

### Follow-up and endpoints

The primary endpoint was a composite of SCD, aborted sudden cardiac death (ASCD), and sustained ventricular tachycardia (VT). SCD was defined as unexpected death either within 1 h of cardiac symptoms in absence of progressive cardiac deterioration, during sleep, or within 24 h of last being seen alive and well. ASCD was diagnosed in patients who received appropriate ICD shock for ventricular arrhythmia or had nonfatal episodes of ventricular fibrillation (VF) or spontaneous sustained VT (>30 s) causing hemodynamic compromise and requiring cardioversion. (For follow-up details, see the Supplemental Appendix).

### Image analysis

**Figure 1:**
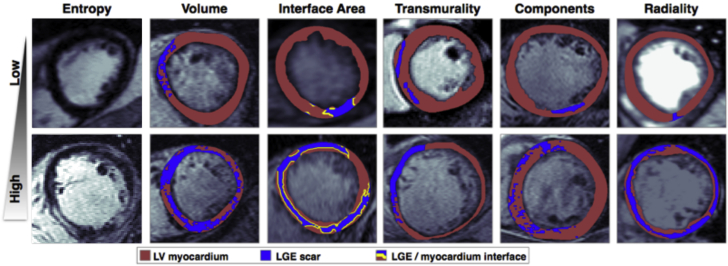
Example of LGE-CMR Images Demonstrating High and Low LGE Metric Values **Red** signifies surviving LV myocardium; **blue** signifies fibrosis; **yellow line** shows interface between surviving myocardium and fibrosis. CMR = cardiac magnetic resonance; LGE = late gadolinium enhancement; LV = left ventricular.

Individual segmented short-axis LGE-CMR images were processed to derive a set of metrics that defined the morphology or texture of the region of fibrosis represented by LGE. LGE metrics were based on measurements of LGE entropy, volume, interface area, transmurality, number of components, and radiality. In particular, the LGE interface area was defined as the total arc-length of the border between myocardium and LGE, multiplied by the slice thickness. The LGE components metric was defined as the total number of 4-connected LGE regions in all slices, and the LGE radiality was defined in each image as the angular variance of all LGE pixels with respect to the center of the blood pool and in each patient as the mean radiality score across slices containing LGE. See the Supplemental Appendix for full details of metric calculations. Figure 1 shows example LGE-CMR images with low (upper row) or high (lower row) values of each of the computed metrics.

### Computational simulations

Segmented images containing LGE were processed as individual short-axis slices and meshed (with computational geometry software Computational Geometry Algorithms Library (CGAL) (https://doc.cgal.org/) into triangular finite element models with maximum edge length 250 μm (9). A monodomain representation was used to simulate electrical activity with the Cardiac Arrhythmia Research Package (10). Conductivities were tuned to match experimentally observed conduction velocities (11), suitably modulated within scar regions (9). Patchy distributions of fibrosis within regions of LGE were represented using the percolation method (9,12, 13, 14).

Simulated programmed electrical stimulation was performed from an endocardial pacing location— consistent with respect to scar in each model (9)—to attempt to induce re-entry; 600 ms activity after the final extrastimulus was simulated, with 10 simulations performed per image slice, testing various scar microstructure combinations (9). For further details, see the Supplemental Appendix.

An additional simulated re-entries metric was derived using the computational simulations. This metric summed the total number of successfully induced re-entries that could be simulated from the 2-dimensional models that were derived from each patient’s LGE short-axis images. Finally, a mechanistic analysis of transmembrane potential dynamics was performed to probe the role of different LGE metrics defining the scar in unidirectional conduction block and re-entry initiation. For further details, see the Supplemental Appendix.

### Statistics

Categorical data are presented as frequency with percentage in parentheses; continuous data are presented as mean ± 1 SD. Comparisons among groups were made using Fisher exact tests for categorical variables and Mann-Whitney tests for continuous variables. Event times were measured from the time of the CMR study. Cox-regression analysis was performed to examine the association between the primary endpoint and LGE shape metrics. Results are presented as hazard ratios (HRs) with 95% confidence intervals (CIs) in SD units. Inverse probability weights (IPWs) were used to adjust for all non-LGE baseline variables (Table 1), along with robust standard errors. For the simulated re-entries the IPW were restricted to 4 covariates (Supplemental Appendix) as the large number of patients with simulated re-entries = 0 prevented us from building IPW for all baseline variables for this variable. The IPWs were generated by solving an optimization problem, which maximally decorrelated the target LGE variable with the potential confounders. All correlations between baseline variables and each LGE metric were reduced to zero following this process. Details regarding the weights and their calculation are provided in the Supplemental Appendix.

**Table 1:** Baseline Patient Characteristics

	Total Cohort (N = 156)	Event-Free (n = 140)	Event (n = 16)	p Value
Age, yrs	56.1 ± 14.5	56.1 ± 14.6	56.2 ± 14.3	0.90
Male	128 (82.1)	117 (83.6)	11 (68.8)	0.17
Body surface area (m^2^)	1.99 ± 0.22	1.99 ± 0.22	1.93 ± 0.23	0.12
Heart rate (beats/min)	74.0 ± 16.4	74.1 ± 16.0	73.2 ± 20.0	0.88
Systolic blood pressure (mm Hg)	123.9 ± 20.5	124.5 ± 20.6	118.2 ± 19.4	0.49
Diastolic blood pressure (mm Hg)	74.0 ± 13.3	74.2 ± 13.5	72.9 ± 11.9	0.57
Diabetes	22 (14.1)	18 (12.9)	4 (25.0)	0.25
Moderate alcohol excess	25 (16.0)	19 (13.6)	6 (37.5)	0.02∗
Smoker (current)	15 (9.6)	13 (9.2)	2 (12.5)	0.65
NYHA functional class				
I	59 (37.8)	55 (39.3)	4 (25.0)	0.003∗
II	70 (44.9)	66 (47.1)	4 (25.0)	−
III/IV	27 (17.3)	19 (13.6)	8 (50.0)	−
Medications				
Beta-blocker	128 (82.1)	114 (81.4)	14 (87.5)	0.74
ACE inhibitor/ARB	137 (87.8)	123 (87.9)	14 (87.5)	1.00
Aldosterone antagonist	72 (46.2)	64 (45.7)	8 (50.0)	0.80
Loop diuretic	86 (55.1)	74 (52.9)	12 (75.0)	0.12
CMR measurements				
LV ejection fraction (%)	36.7 ± 11.8	36.9 ± 11.8	35.6 ± 12.4	0.72
LV end diastolic volume index (ml/m^2^)	139.3 ± 38.9	139.1 ± 39.0	140.6 ± 38.4	0.93
LV mass index (g/m^2^)	100.9 ± 30.2	99.2 ± 27.3	115.3 ± 47.4	0.21
RV ejection fraction (%)	51.6 ± 13.8	51.8 ± 13.6	50.2 ± 15.5	0.80
LGE metrics				
Interface area (cm^2^)	103.1 ± 78.6	99.4 ± 76.7	135.0 ± 90.0	0.06
Volume (cm^3^)	9.7 ± 9.3	9.4 ± 9.2	12.5 ± 10.4	0.05
Entropy	3.63 ± 0.44	3.63 ± 0.44	3.71 ± 0.46	0.32
Mean transmurality	0.39 ± 0.11	0.39 ± 0.11	0.42 ± 0.08	0.12
Number of components	7.4 ± 3.3	7.4 ± 3.5	7.9 ± 2.9	0.43
Mean radiality	0.35 ± 0.18	0.35 ± 0.18	0.40 ± 0.18	0.22
Simulated re-entries	0.7 ± 1.8	0.5 ± 1.4	1.8 ± 3.7	0.06

Values are mean ± SD, compared with Mann-Whitney tests, or n (%), compared with Fisher exact tests.

ACE = angiotensin-converting enzyme; ARB = angiotensin receptor blocker; CMR = cardiac magnetic imaging; LGE = late gadolinium enhancement; LV = left ventricular; NYHA = New York Heart Association.

∗ Statistically significant, p < 0.05.

Multivariate survival analysis was also performed, including common clinical covariates currently used to risk stratify this group of patients (New York Heart Association [NYHA] functional class and LVEF Model 1), as well as ICD placement, considered as a time-varying covariate (Model 2).

Finally, a risk-stratification scheme was demonstrated by dividing patients into terciles of low, medium, and high LGE interface areas, with each tercile containing one-third of the patients. HRs with 95% CIs were calculated from a multivariate IPW-adjusted Cox model, and Kaplan-Meier survival curves were estimated and compared with a log-rank test. Statistical analysis was performed using SPSS Statistics (IBM, 2017:v25, Armonk, New York) for comparison of baseline variables, and using the Python package Lifelines for survival analysis; p < 0.05 was deemed statistically significant throughout.

## Results

### Baseline characteristics and device implantations

Of the 156 patients included, 128 (82.1%) were men, with mean age 56.1 ± 14.5 years and mean LVEF 36.7 ± 11.8. At baseline, 87.8% were on angiotensin converting enzyme (ACE) inhibitors or angiotensin receptor blockers (ARBs), 82.1% on beta-blockers, 46.2% on mineralocorticoid receptor antagonists (MRAs) (aldosterone antagonist), and 55.1% were on diuretics. Considering LGE metrics, mean entropy was 3.63 ± 0.44; mean volume was 9.7 ± 9.3 cm^3^; mean interface area was 103.1 ± 78.6 cm^2^; mean transmurality was 0.39 ± 0.11; mean number of components was 7.4 ± 3.3; and, finally, mean radiality was 0.35 ± 0.18. The mean number of simulated re-entrant activations (in all imaging slices of a patient) was 0.7 ± 1.8. Other baseline patient characteristics are presented in Table 1.

Over the course of follow-up, 56 patients (35.9%) received ICDs before the occurrence of the primary endpoint, 38 (24.4%) of them also received cardiac resynchronization therapy. Fifty-five patients received ICDs for primary prevention of SCD. Individual cases with risk factors for SCD that did not meet primary prevention guidelines were discussed within a multidisciplinary team meeting. A single patient received a device for secondary prevention following an arrhythmic episode before baseline. Further details regarding ICD implantation times can be found in the Supplemental Appendix.

### Primary endpoint: occurrence of arrhythmic event

During a median follow-up time of 1,611 (interquartile range [IQR]: 881 to 2,341) days, 16 patients (10.3%) met the primary arrhythmia endpoint, with 11 of these patients receiving ICD placements after baseline. The median time to event was 1,639 (IQR: 845 to 2,433) days. The cohort was divided into groups based on the occurrence of the primary endpoint (Table 1, columns 3 to 5). Patients who met the primary endpoint were more likely to drink excessively (p = 0.02) and have higher NYHA class (p = 0.003).

### Time-to arrhythmic event analysis of the LGE shape metrics

In univariate time-to event analysis (Table 2), the only LGE shape metric associated with the primary endpoint was interface area (HR: 1.55; 95% CI: 1.06 to 2.28; p = 0.025). Following IPW adjustment to account for the non-LGE confounding variables, interface area remained significantly associated with the primary endpoint (HR: 1.75; 95% CI: 1.24 to 2.47; p = 0.001), and LGE volume became significant (HR: 1.44; 95% CI: 1.07 to 1.94; p = 0.015). In multivariate analysis (Table 3), following adjustment for LVEF and NYHA Class (Model 1), interface area remained associated with the primary endpoint (HR: 1.57; 95% CI: 1.06 to 2.33; p = 0.02). A further multivariable model (Model 2) was constructed as part of a sensitivity analysis, adjusting for ICD placement as a time-varying covariate. Following adjustment, interface area again remained associated with the primary endpoint (HR: 1.63; 95% CI: 1.07 to 2.49; p = 0.02).

**Table 2:** Univariate Time to Arrhythmic Event Regression Results

LGE Metrics†	Unadjusted Univariate			IPW Adjusted Univariate‡		
	HR	95% CI	p Value	HR	95% CI	p Value
Interface area	1.55	1.06–2.28	0.025†	1.75	1.24–2.47	0.001∗
Volume	1.37	0.96–1.95	0.087	1.44	1.07–1.94	0.015∗
Entropy	1.56	0.95–2.58	0.081	1.39	0.68–2.84	0.36
Mean transmurality	1.30	0.85–1.97	0.223	1.42	0.99–2.05	0.06
Number of components	1.05	0.68–1.63	0.819	1.14	0.76–1.71	0.53
Mean radiality	1.35	0.84–2.17	0.212	1.48	0.97–2.26	0.07
Simulated re-entries	1.39	1.11–1.75	0.004∗	1.40	1.23–1.59	<0.001∗

CI = confidence interval; HR = hazard ratio; IPW = inverse probability ratio; LGE = late gadolinium enhancement.

∗ Statistically significant, p< 0.05.

† In standardized units (per SD).

‡ Adjusted using inverse probability weighing (IPW) for baseline variables.

**Table 3:** Multivariate Time to Arrhythmic Event Regression Results

	HR	95% CI	p Value
Model 1			
LGE interface area†	1.57	1.06–2.33	0.02∗
LV ejection fraction†	1.01	0.97–1.06	0.67
NYHA functional class III/IV	5.83	2.15–15.78	<0.001∗
Model 2			
LGE interface area†	1.63	1.07–2.49	0.02∗
ICD receipt‡	5.19	1.81–14.94	0.002∗

CI = confidence interval; HR = hazard ratio; LGE = late gadolinium enhancement; LV = left ventricular; NYHA = New York Heart Association.

∗ Statistically significant, p < 0.05.

† In standardized units (per standard deviation [SD]).

‡ As a time-varying covariate.

**Figure 2:**
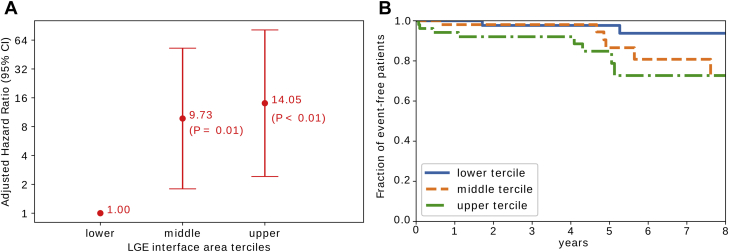
LGE Interface Area Tercile-Based Arrhythmia Risk-Stratification Scheme **(A)** Forest plot showing relative hazard ratios for LGE interface area terciles (lower <60 cm^2^, middle 60 to 122.69 cm^2^, upper ≥122.70 cm^2^) calculated by a Cox-regression model with inverse probability adjustment for baseline covariates. **(B)** Kaplan-Meier survival curves for patients in the upper, middle, and lower LGE interface area terciles. CMR = cardiac magnetic imaging; LGE = late gadolinium enhancement.

Finally, we repeated the IPW-adjusted analysis with the interface area divided into terciles (Figure 2A). The number of patients meeting the primary endpoint increased with each tercile, with 2, 6, and 8 events in the lower, middle, and upper terciles, respectively, giving respective event-rates of 3.8%, 11.5%, and 15.4%. The results of the IPW analysis show that patients in the middle (60 to 122.69 cm^2^) and upper terciles (>122.70 cm^2^) of LGE interface area were at significantly greater arrhythmic risk (middle tercile HR: 9.73; 95% CI: 1.80 to 52.71; p = 0.01, upper tercile HR: 14.05; 95% CI: 2.41 to 81.74; p < 0.01), than those in the first. An additional Kaplan-Meier analysis (Figure 2B) confirmed that patients in the upper tercile of interface area were more likely to experience events than those in the lower tercile (p = 0.02).

### Simulated re-entries

A total of 6,990 stimulation protocol simulations were performed. This resulted in 102 simulated re-entries in 32 patients, representing 5.7% of the 1,800 simulations in the patients with re-entries. Six patients with simulated re-entries also met the arrhythmic endpoint during follow-up. Both the event-rate (6 of 32, ≈18.8% vs. 10 of 124, ≈8.1%) and mean interface area (149.2 cm^2^ vs. 91.2 cm^2^, p < 0.001) were greater for patients with simulated re-entries than for patients with no simulated re-entries. The number of simulated re-entries was associated with the primary endpoint in univariate analysis (HR: 1.39; 95% CI: 1.11 to 1.75; p < 0.01) and also after IPW adjustment (HR: 1.40; 95% CI: 1.23 to 1.59; p < 0.01).

### Uncovering the proarrhythmic mechanism of LGE interface area using computational modeling

**Figure 3:**
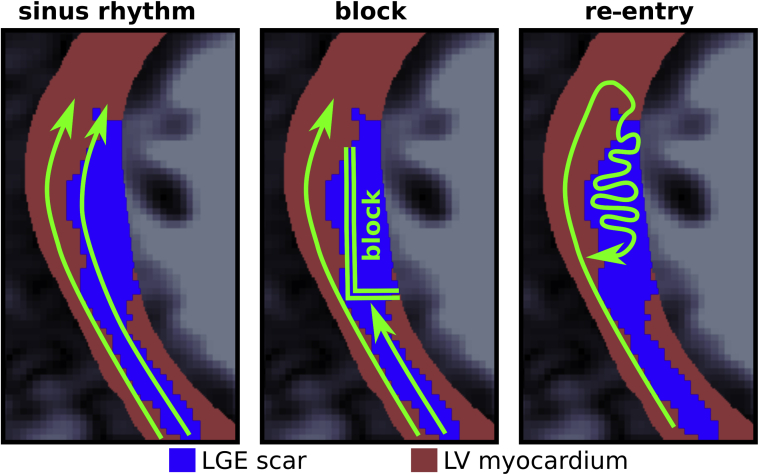
Illustration of the Block and Re-Entry Mechanism Seen in the Computational Simulations Images are a close up of an area of LGE in an example segmented CMR scan. **(Left)** Areas of LGE are initially permeable to electrical activation waves under sinus rhythm. **(Center)** Lines of block develop along the LGE interface and within the LGE under faster pacing rates. **(Right)** The LGE is activated by a retrograde path, and the wavefront eventually escapes the LGE zone to reactivate the neighboring myocardium. CMR = cardiac magnetic imaging; LGE = late gadolinium enhancement

An in silico approach was used to elucidate mechanistic explanations for the importance of scar–myocardium interface upon increased arrhythmia susceptibility. Figure 3 presents a sketch of the re-entry mechanism observed in the 2-dimensional simulations. A key feature is the presence of an LGE interface, internal to the myocardium, which leads to LGE and normal myocardium coexisting side by side. Under sinus-rhythm pacing, the LGE regions are permeable to electrical activation waves (Figure 3, left). When the pacing rate is increased, the restitution properties of the LGE tissue, in combination with fibrosis, slow down conduction and eventually lead to lines of block, which are often aligned with the LGE interface (Figure 3, center). Electrical propagation in the non-LGE areas is however still viable, and a wave enters the area of transient conduction block via a back-door path. The wavefront then meanders within the LGE until the surrounding tissue regains excitability, at which point a re-entrant activation takes place (Figure 3, right).

**Figure 4:**
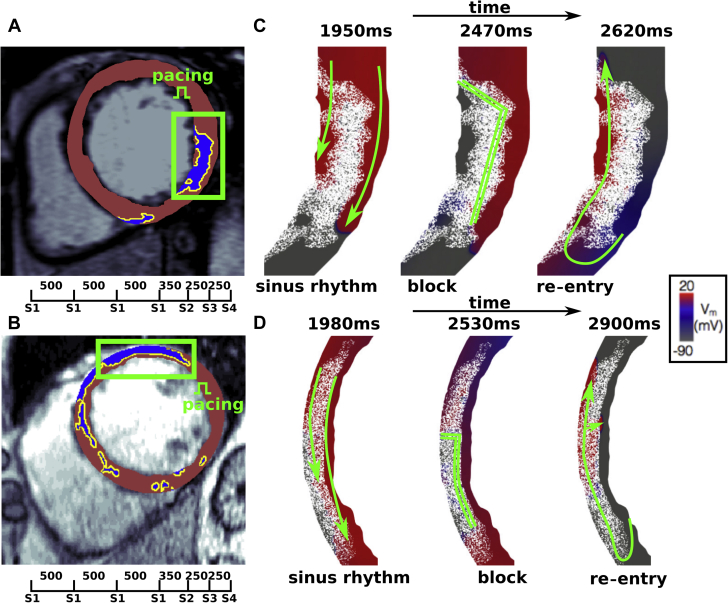
Two Examples of Computational Simulations Demonstrating Block and Re-Entry **(A,B)** Source LGE-MRI images. **(C,D)** Time snapshots of the simulated transmembrane potential (V_m_) generated by the 2-dimensional computational models.

In Figure 4, we present time snapshots of the transmembrane potential from 2 computational simulations displaying the described re-entrant mechanism. In both cases, the LGE zones are initially permeable under sinus rhythm but facilitate a block and re-entry with an increased pacing rate.

**Figure 5:**
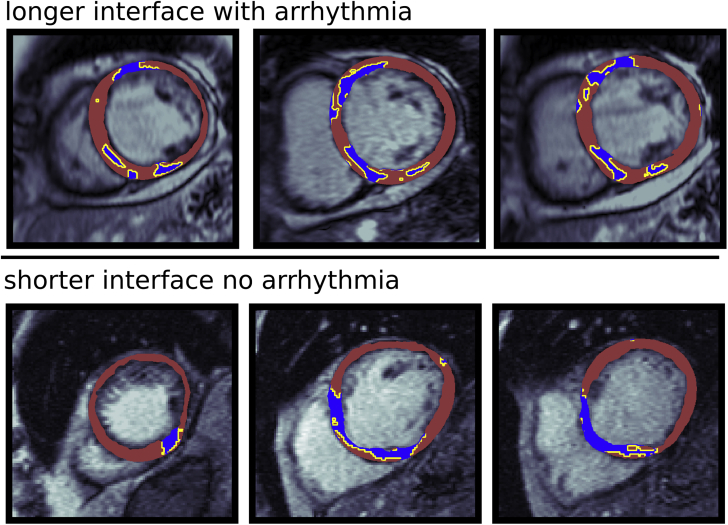
Example LGE-MRI Slices From 2 Patients With Similar LGE Volumes but Differing Interface Areas and Outcomes The LGE volumes of the 2 patients are similar (**top:** 14.5 cm^3^, **bottom:** 14.0 cm^3^), but the interface areas (**top:** 156.3 cm^2^, **bottom:** 86.8 cm^2^) and outcomes (**top** reached arrhythmic endpoint; **bottom** did not) differ. LGE = late gadolinium enhancement; MRI = magnetic resonance imaging.

The importance of the interface of the scar, above its volume, is further emphasized in Figure 5, which shows 2 patients with similar volumes of LGE (14.0 and 14.5 cm^3^) but very different interface areas. The patient with the longer interface area (top, 156.3 cm^2^) reached the primary endpoint of SCD, whereas the patient with the lower interface area (bottom, 86.8 cm^2^) did not. Figure 5 (top) shows long contiguous lengths of myocardial–LGE interface, at which re-entrant activations may occur according to the mechanisms that we have simulated. In contrast to this, the myocardial-LGE interface in Figure 5 (bottom) is shorter. Indeed, the patient with the higher interface area had 2 re-entries successfully induced in the corresponding in silico models compared with none in the patient with the lower interface area.

## Discussion

We have shown that a novel LGE-CMR–based metric, which quantifies the area of interface between fibrotic tissue and surrounding surviving myocardium, is associated with major arrhythmic events in a well-characterized cohort of patients with NIDCM and mid-wall LGE. Detailed computational modeling has further provided plausible a mechanistic description of the role that such a region of tissue transition might have in promoting unidirectional conduction block and the formation of re-entrant circuits, corroborating our findings. LGE volume was also significantly associated with the primary endpoint in an IPW-adjusted analysis, albeit with a lower HR than interface area. Other morphological LGE metrics quantifying scar entropy, transmurality, number of components, and radial extent were not associated with the primary endpoint.

### LGE interface area as an independent predictor of arrhythmia

In ischemic cardiomyopathy (ICM), border-zone regions define areas of patchy fibrosis of intermediate LGE surrounding compact scar (15,16). Such areas are known to provide an arrhythmogenic substrate that facilitates conduction block and re-entry around a necrotic core (12). Border-zone volume on LGE-CMR has also been shown to independently predict arrhythmic events and SCD in post-MI patients (15,16). However, in NIDCM there is no corresponding definition of border-zone, largely because of the lack of any direct histological evidence (17). The fibrotic architecture in NIDCM tends rarely to be compact but more typically patchy or diffuse throughout (4,17). Thus, differentiating LGE in NIDCM into 2 separate regions (core and border-zone) may not be appropriate and has not shown to be predictive of ICD therapy in this population (16).

As an alternative to scar border-zone, we considered the interface between fibrotic areas and surrounding surviving myocardium. Such an interface represents an abrupt transition in electrical properties such as local electrotonic loading, conduction velocity, potential myofibroblast coupling, and electrical remodeling. Once the scar itself is defined, the interface length (on a single image slice) has a robust and consistent definition (Figure 1), representing the perimeter around the scar, which is internal to the myocardium. This is in contrast to border-zone delineation, which is subject to many different definitions and methods of computation (16,17). Although related to total fibrosis volume and other metrics, interface area still represents a fundamentally distinct image-based metric. For example, 2 different regions of mid-wall scar with characteristic striated patterns (with the same LGE volume) may have very different interface areas, depending on whether the identified scar is in contact or not with the epi or endocardial surface; the case in contact would have a reduced interface area.

### Mechanism of arrhythmogenesis by LGE interface region

We have corroborated our empirical observation of the association of interface area of LGE in NIDCM with major arrhythmic events by using detailed computational simulations to dissect possible mechanisms by which these interface regions may facilitate the genesis of reentrant activity. In silico analysis showed that conduction block of a premature beat can frequently occur as the electrical wavefront attempts to pass from the healthy area to the fibrotic region. Furthermore, isolated regions of block can occur deep within a scar as wavefronts attempt to navigate through tortuous pathways formed by patchy fibrosis. Such unidirectional conduction block at fast pacing rates due to complex fibrotic pathways has been demonstrated via computational simulations in other pathologies (12,13) and in our previous work in NIDCM (9,14). Here, we elucidate the specific importance of the transition between impaired conduction in the fibrosis and faster conduction within the surrounding tissue in NIDCM.

The identified re-entry mechanism can occur transiently and depends on a number of competing factors such as wavefront direction, morphology, pacing rate, as well as the specific microfibrosis pattern that governs the balance between electrotonic current source and sink (13,14). Such patterns are effectively random, and therefore longer interface lengths and areas increase the likelihood that a particular interface region will provide the suitable substrate to cause isolated unidirectional block. Furthermore, wavefronts that are trapped in the LGE behind a line of block must survive until the surrounding healthy myocardium has regained excitability to cause a re-entry. Longer interface areas may facilitate this process by extending the potential path length between a wave’s entry and exit sites within the LGE, thereby extending the potential life-span of wavefronts within the LGE and making re-entries more likely to occur.

Finally, we emphasize that our modeling suggests a potential mechanism for the genesis of the initial reentrant activity; whether these initial re-entrant waves remain monomorphic or degenerate into polymorphic VT/VF may depend on a number of other factors that we have not investigated.

### LGE entropy

Texture analysis of LGE-CMR images has been performed by 2 recent works within nonischemic cohorts, with differing results regarding its association with arrhythmic events (6,18). Here, we computed entropy using the native approach of Muthalaly et al. (6) but focused only on the enhanced region—as in Gould et al. (18)—as opposed to the entire LV as in Muthalaly et al. (6). Although there was a trend toward an association between LGE entropy and primary endpoint in our study, this did not reach statistical significance. Further investigations into the role of LGE entropy are therefore required.

### Other LGE metrics

The total fibrosis extent in NIDCM has been shown to be predictive of adverse arrhythmic events (2,16), with a more recent work showing a nonlinear relationship between fibrosis burden and likelihood of arrhythmia, so that only a small amount of fibrosis is required to cause a large increase in arrhythmia susceptibility (5). Our results for LGE volume are therefore in agreement with these previous works, as we observed a trend toward higher baseline LGE volume in patients with arrhythmic events as well as a statistically significant HR in the adjusted time-to event analysis (Central Illustration).

**Central Illustration:**
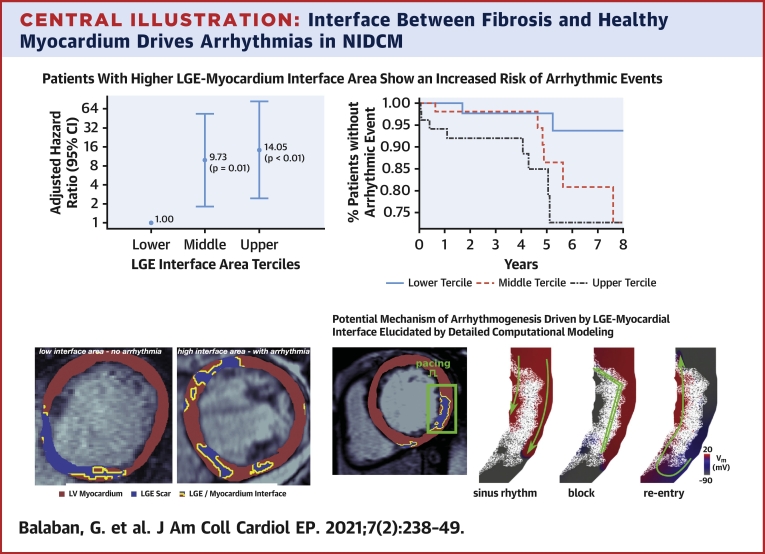
Interface Between Fibrosis and Healthy Myocardium Drives Arrhythmias in NIDCM Patients in the highest tercile of LGE interface (>122.70 cm^2^) show a significantly elevated risk of arrhythmias, shown in Cox-regression analysis (forest plot top, **left**) and Kaplan-Meier survival analysis **(top, right)**. Image-based computational simulations provide an important tool to elucidate mechanistic understanding of how fibrosis-myocardium interface facilitates arrhythmogenesis in this patient population **(bottom)**. LGE = late gadolinium enhancement; LV = left ventricular.

Our examination of the number of LGE components and mean radiality was partially motivated by the characteristic arrhythmogenic “stria” pattern of LGE seen in competitive athletes (8) and displayed in our simulated re-entry mechanism (Figure 3), with fewer connected LGE components spanning a potentially high radiality. The lack of predictive power of the components and radiality metrics suggests that other LGE metrics better represent the arrhythmogenic substrate provided by such a stria pattern. For example, if such stria patterns “touch” the epi or endocardial surface boundaries, this does not affect the metrics of number of components or radiality, but it would significantly reduce the interface value and potential arrhythmic risk in light of our uncovered mechanisms (Figure 3).

### Arrhythmia risk stratification with computational simulations

The number of reentrant activations generated by our computational simulations showed an association with real-life arrhythmic events. This serves as the first proof-of-concept for using computational simulations for arrhythmic risk stratification in patients with NIDCM. Such computational simulations have successfully been applied to risk stratifying patients with ICM (19). In the nonischemic setting, further improvements in prediction of risk may be possible by considering 3-dimensional simulations (20), a wider variety of pacing locations, and personalized electrophysiological properties.

### Study limitations

Limitations associated with our CMR imaging have been fully described in our previous study (5). Briefly, parametric imaging (capable of identifying regions of diffuse fibrosis) was not used, as it was not available at the outset of the study. Although different contrast agents were used, there was no evident difference in the quantity, pattern, or location of LGE for patients scanned with gadobutrol compared with gadopentetate dimeglumine. Furthermore, delineation of scar tissue in NICM is known to be challenging because of the heterogeneity in fibrosis architecture compared with patients with ICM (17), as well as intraslice variability in peak voxel signal intensity. To limit peak intensity variability, a standardized imaging protocol was used, and fibrosis was only classified in a particular slice when there was a clear and reproducible area of late enhancement indicative of replacement fibrosis.

Segmentation of fibrosis in NIDCM can be performed with a number of different computational methods (16,17), which may affect the morphology of the scars and quantitative LGE-derived metrics used here. Here, we employed a semiautomated FWHM method in which only reproducible LGE was accepted by the analysts who were blinded to the patient outcomes. We believe that FWHM is the most widely used method, and have previously reported good interobserver reproducibility (5).

We recognize the modest number of events within the population. Nevertheless, we identify a strong and robust association between interface area and a clinically meaningful endpoint, which is supported by detailed mechanistic computational modeling. Overall, the data suggest that quantification of interface area has the potential to improve clinical decision making for patients with NIDCM and LGE. The observational design of our study meant that we could not rule out the influence of unmeasured confounding. However, we were able to adjust for measured baseline covariates using the IPW methodology. Given the possibility that there was detection bias related to placement of ICDs, we adjusted for time-varying ICD presence in a multivariate model, and the overall results remained qualitatively similar.

## Conclusions

Interface area, defining the surface between fibrotic scar and healthy myocardium, is associated with major arrhythmic events in NIDCM, along with the number of simulated re-entries and LGE volume. The novel interface area metric provides an alternative to border-zone analysis, which has been shown to be unreliable in predicting arrhythmic events in this population. The mechanistic link between scar–myocardium interface and arrhythmogenesis uncovered from in silico analysis supports the clinical relevance of our findings.

Perspectives **COMPETENCY IN MEDICAL KNOWLEDGE:** The interface area between healthy myocardium and LGE on CMR is associated with arrhythmic events in patients with NIDCM and LGE. Mechanistically, functional block along the interface may facilitate re-entry, and such events can be simulated for a patient with computational electrophysiology models.

**TRANSLATIONAL OUTLOOK:** Our findings suggest that quantification of LGE–myocardium interface area and re-entry simulations from computational electrophysiology models may be useful additional tools to identify patients with fibrosis at highest risk of arrhythmia. Future modeling efforts should consider 3-dimensional models and personalized electrophysiological properties.

## Funding Support and Author Disclosures

This research was primarily supported through a New Investigator Research Grant to Dr. Bishop from the Medical Research Council (MR/N011007/1). The research was further supported by the National Institute for Health Research (NIHR) Biomedical Research Centre (BRC) and CRF based at Guy’s and St Thomas’ NHS Foundation Trust and King’s College London, along with Imperial College London NIHR BRC, The Royal Brompton CRC and NIHR BRU, Alexander Jansons Foundation, and the BHF (FS/15/29/31492). The views expressed are those of the authors and not necessarily those of the NHS, the NIHR, or the Department of Health. This work was also supported by the Wellcome EPSRC Centre for Medical Engineering at King’s College London (WT 203148/Z/16/Z). All authors have reported that they have no relationships relevant to the contents of this paper to disclose.
