# Supplementary material for: Late-Gadolinium Enhancement Interface Area and Electrophysiological Simulations Predict Arrhythmic Events in Patients With Nonischemic Dilated Cardiomyopathy
Source: JACC Clin Electrophysiol. 2021 Feb;7(2):238–49. doi: 10.1016/j.jacep.2020.08.036 (PMC7900608; doi:10.1016/j.jacep.2020.08.036)
Supplement: Supplemental Data [file mmc1.pdf]

Supplemental Material

Study Population

Patients referred to Royal Brompton Hospital for CMR for the evaluation of NIDCM between 2006 and 2015 *and who had evidence of mid-wall or subepicardial LGE* (characteristic of non-ischemic cardiomyopathy) were included. As the goal of our work was to investigate the effect of LGE shape upon arrhythmic risk, all patients without LGE were excluded from our analysis. Furthermore, all patients with evidence of sub-endocardial LGE (characteristic of ischemic cardiomyopathy) were also excluded. The eligible patients were enrolled into our Cardiovascular Biobank Project, designed to facilitate streamlined, collaborative inter-institutional work. All patients gave written informed consent, the study was approved by the National Research Ethics Committee and was performed in accordance with the Declaration of Helsinki. Eligible patients had a diagnosis of DCM confirmed by an independent consultant cardiologist based on clinical details and CMR findings. All patients had increased left ventricular end-diastolic volume indexed to body surface area and reduced LVEF compared with published reference ranges normalised for age and sex[1]. Exclusion criteria have been comprehensively described elsewhere[2] and included ischemic heart disease defined as stenosis >50% in a major coronary artery, inducible ischemia on functional testing or subendocardial or transmural LGE indicative of previous myocardial infarction. Only patients with clear and reproducible areas of LGE were included. Other forms of cardiomyopathy, including sarcoidosis, acute myocarditis and amyloidosis were excluded either at time of referral or following CMR.

The baseline age of two patients was missing, and the mean age of the other patients was imputed for these two patients.

Follow-Up

Follow-up was performed using postal questionnaires and accessing information from the patients' physicians and hospital notes. All events were adjudicated by an independent committee blinded to

the CMR results. Mortality status was verified from the UK Health and Social Care Information Service. Cause of death was established from death certificates, postmortem and medical records.

Image Analysis

Entropy was measured by the standard Shannon Entropy[3], being applied to the native grey-scale image within the segmented fibrosis region[4], providing a measure of fibrotic disorder present. Units: dimensionless.

Volume was computed by summing together all pixels that contained LGE multiplied by slice thickness. Units: cm^3.

Interface Area was measured by extracting the border between myocardium and LGE and adding together every borders' arclength multiplied by slice thickness. Note that the interface between the scar and the valvular annuli was not considered, neither was the interface between scar and epicardial or endocardial surfaces.

Units: cm^2

Transmurality was measured by a ray tracing method in which 580 rays emanated from the central pixel of the blood pool. The slice-specific transmurality score was then defined to be the mean fraction of LGE along each ray that intersected scar, while the patient's transmurality score was the mean of the slice-specific scores among slices with LGE. Units: dimensionless.

Number of Components was computed by a connected component algorithm, defined to be the total number of 4-connected regions of LGE in all slices.

Radiality was measured in each slice as the angular variance of all LGE pixels with respect to the central blood pool pixel, and for each patient as the mean score of the slices containing LGE. Units: normalised angle (radians/$2\boldsymbol{\pi}$).

All metrics were computed with an in-house Python script which is freely available at www.github.com/GabrielBalabanResearch/lgemri_scar_metrics. Figure S1 shows histograms of all computed LGE metrics across the full patient cohort.

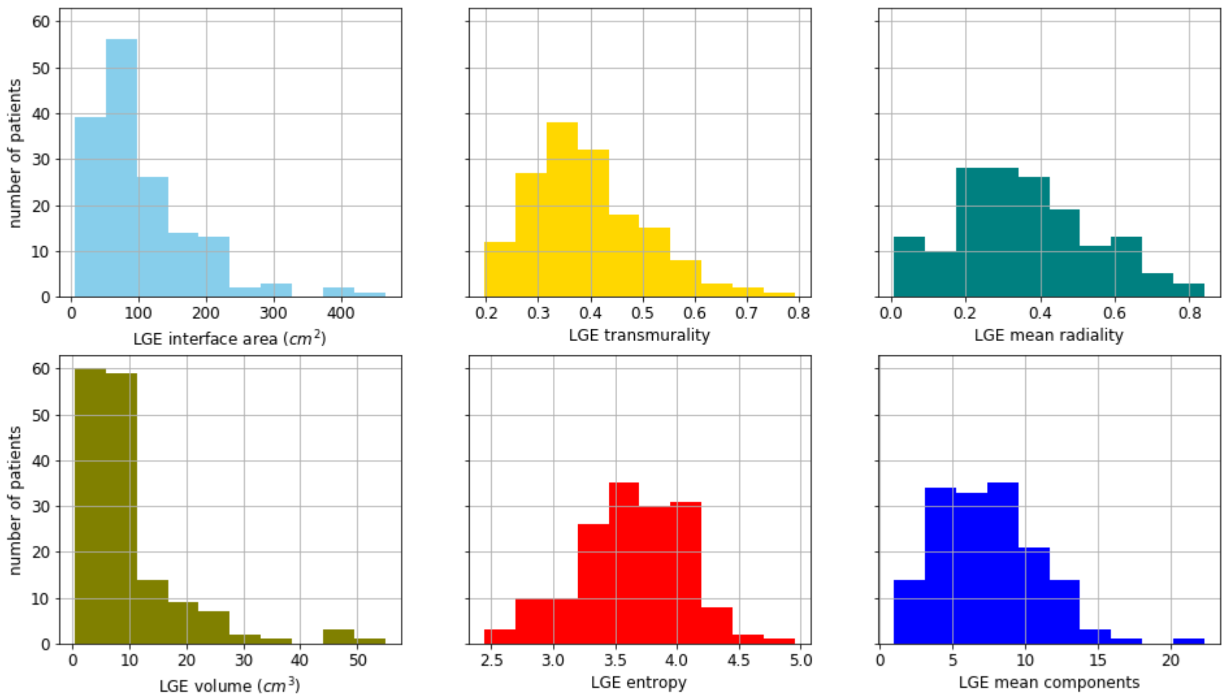

Figure S1: Histograms showing computed LGE metrics across the patient cohort.

Calculation of the Inverse Probability of Weights

Inverse probability weights (IPW) were calculated for each LGE metric using a non-parametric method[5]. In brief the method solves an optimization problem that generates a set of weights which maximally de-correlate the target variable with the potential confounders, while maximizing the empirical likelihood of observing the data. We implemented the IPW method in Python and used the L-BFGS-B solver from Scipy to solve the optimization problem. Our potential confounder set consisted of all baseline variables listed in Table 1 (main article) that were not LGE metrics, as well as ICD or CRT receipt during follow-up. Figure S2 shows the distributions of the resulting IPW; the mode of each distribution is very close to 1, and most weights have value less then 4. In Figure S3 we

visualise the Pearson correlations between the LGE metrics and other baseline variables. All correlations were reduced to 0 after reweighing.

For the simulated reentries variable, it was not possible to calculate IPW using all baseline variables due to the large number of patients (124) with simulated reentries = 0. Instead, we focused on a limited set of the most important confounders, moderate alcohol excess, and NYHA class (which were significantly different in the event and non-event patient groups) and ICD receipt. The distribution of the simulated reentry variable, as well as it's IPW and Pearson correlations are shown in Figure S4.

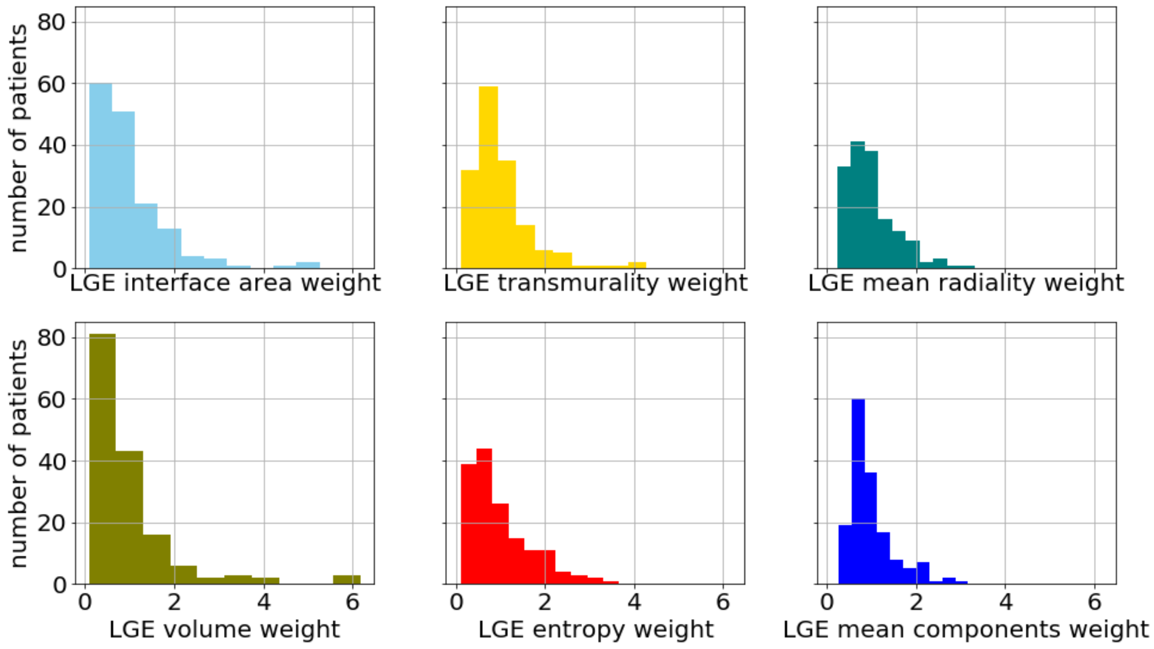

Figure S2: Distributions of inverse probability weights for each LGE metric.

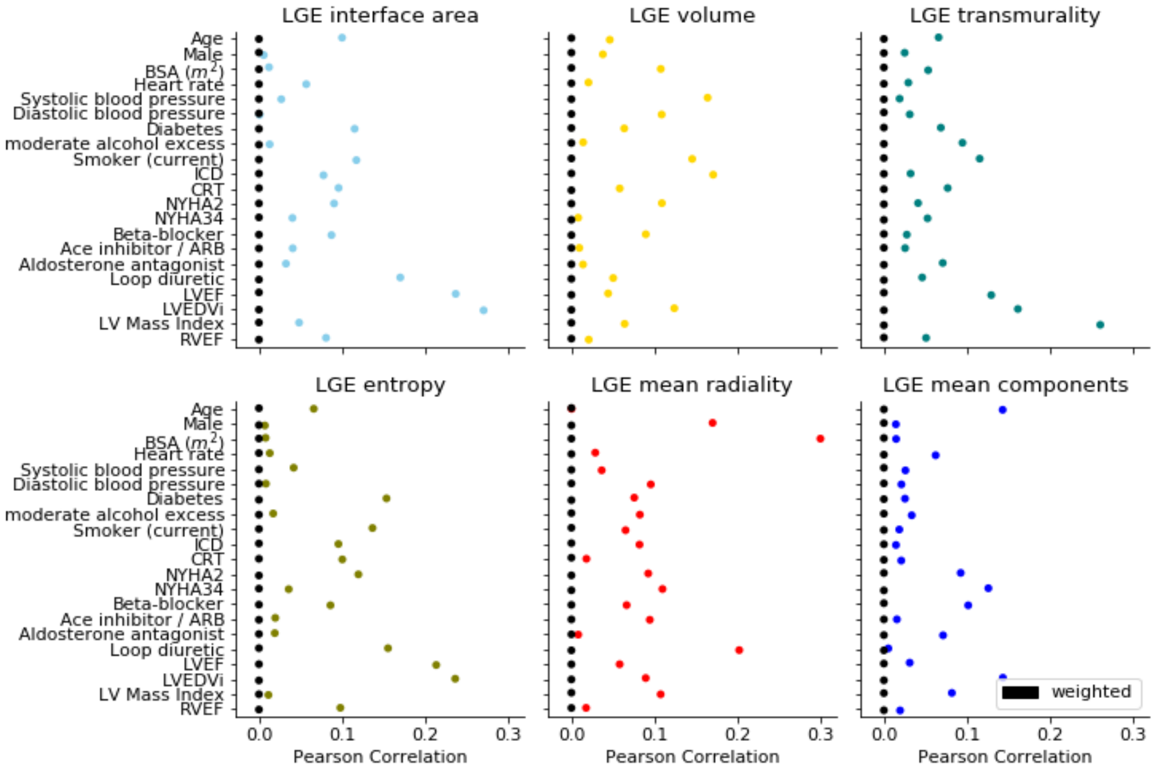

Figure S3: Correlations between LGE metrics and other variables before and after inverse probability weighing.

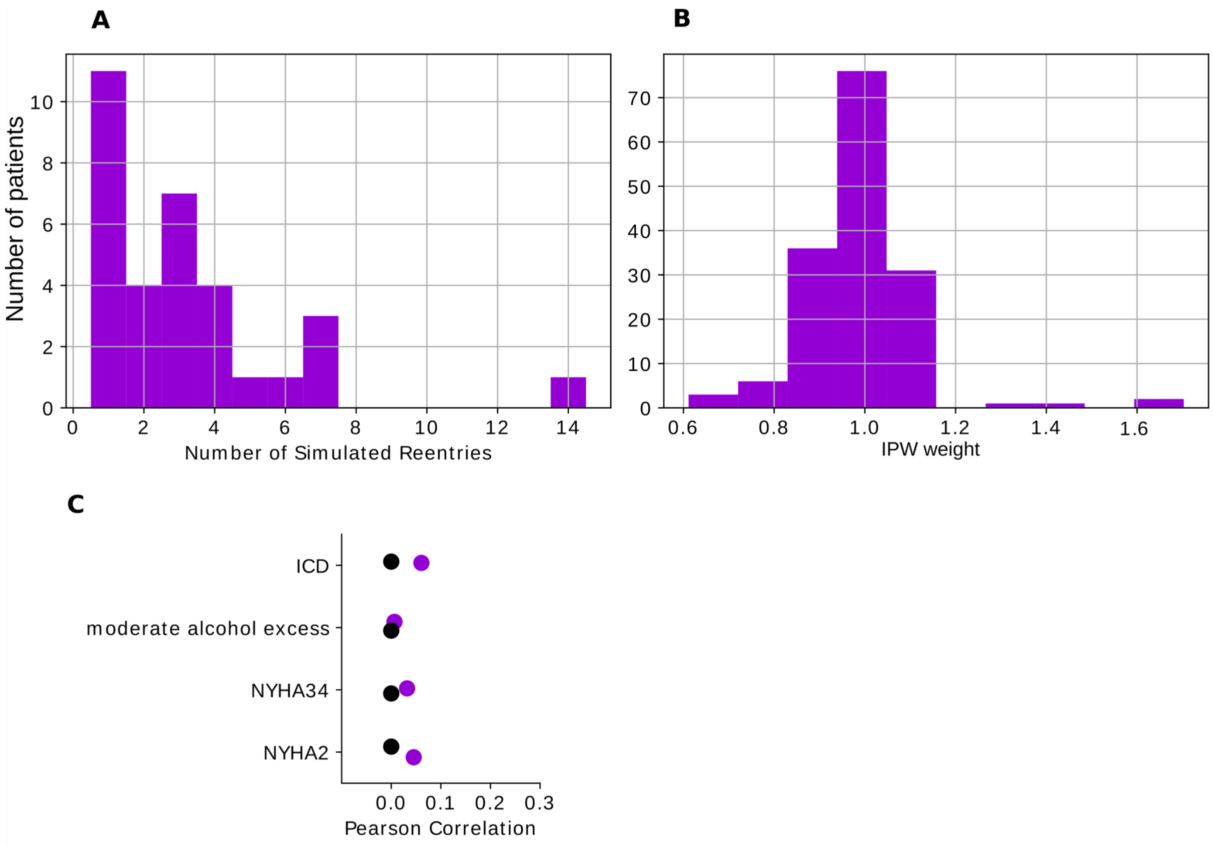

Figure S4 A) Histogram of the number of simulated reentries in the 32 patients with simulated reentries >0. **B)** Histogram of simulated reentry IPW based on ICD, moderate alcohol excess, and NYHA class. **C)** Pearson correlations of variables with simulated reentries before (purple) and after (black) reweighing.

Data on ICD Implantation

Figure S5 shows the time-course of ICD implantation (blue curve) along with the time-course of patients reaching the primary endpoint (red curve). As can be seen from the figure, approximately half of all ICD implantations occurred within 6 months after baseline; however, approximately half of all recorded primary events occurred within 4 years of follow-up.

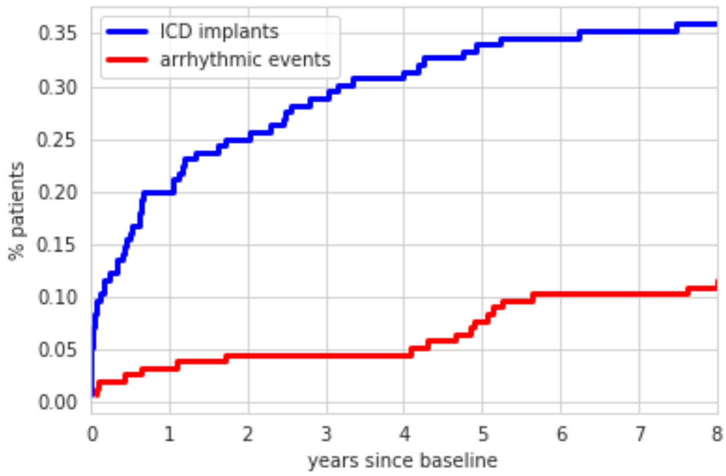

Figure S5: Fraction of total patient cohort receiving ICD (blue curve) or reaching the primary endpoint (red curve) as a function of time during follow-up.

Computational Simulations

Computational simulations were performed as part our previous study on 2D scar shapes and microstructures[6]. In summary, our method for running computational simulations was as follows: segmented images containing LGE were processed as individual short-axis slices and meshed (computational geometry algorithms library CGAL: https://doc.cgal.org/) into triangular finite element models with maximum edge length 250um. A monodomain representation was used to simulate electrical activity with a human ventricular cell model[7] implemented in the Cardiac Arrhythmia Research Package[8]. Realistic myo-fibre architecture was incorporated using a rule-based method[9]. Conductivities were tuned to match experimentally observed conduction velocities[10], with conduction suitably modulated within scar regions, as performed in our previous study[6].

Patchy distributions of fibrosis were represented using the widely-used percolation method[6,11]. Briefly, areas defined as scar in image segmentations had triangular elements or mesh edges randomly removed, with a probability that depended on the normalized local image intensity on the LGE image.

In this manner, less compact regions of fibrosis (characteristic of NIDCM[12]), could be incorporated into the models, allowing conduction of activation waves through the scar substrate, albeit via slowed and convoluted pathways [13].

Simulated programmed electrical stimulation was performed from an endocardial pacing location determined to be in a consistent location with respect to scar in each model (obtained by minimizing a cost function[6]). A steady-state train of 4 S1 pulses at 500ms coupling intervals was followed by up to 5 shorter extra-stimuli, the timing of each determined by the local effective refractory period (ERP). Following each stimulus sequence, 600ms of activity was simulated and any reentrant transmural reentrant activations were recorded, that is activation waves which reversed direction and reactivated the original pacing location. A detailed analysis of activation wavefront dynamics was performed to identify the mechanistic role of different LGE metrics defining the scar in unidirectional conduction block and reentry initiation. A total of 10 simulations were run per image slice, testing 10 scar microstructure combinations, with 5 different scar density levels and 2 fibrosis types (interstitial and replacement fibrosis represented by mesh triangle and mesh edge removal respectively), for further details see [6]. The pacing location, and assignment of electrical and ionic properties to tissues, were kept constant in each image slice.

Detailed Mechanistic Descriptions of the Example Computational Simulations of Reentry

Figure 4 in the main manuscript shows 2 simulations highlighting the specific role of the scar-myocardial interface in the genesis of reentry. Below, we provide a more in-depth description of these simulations.

Simulation 1: Panel C (1950ms) shows the wavefront 100ms following the final S1 beat. Here, the wavefront passes easily through the healthy myocardium at the epicardial and endocardial tissue edges (as the scar is largely mid-myocardial in this example). However, activation of the scar itself does occur, albeit greatly delayed due to the tortuous conduction pathways the wave is forced to take

through the fibrotic tissue. The panel C (2470ms) shows the wavefront 120ms after the final S4 of the stimulus train. Due to the reduced diastolic interval, conduction blocks at along the interface shown by the green parallel lines. However, it continues to propagate along the healthy myocardium in the sub-epicardial region, and conduction occurs (albeit slowly) across the interface in this area, as before. Panel C (2620ms) shows that as activation propagates slowly through the scar, it reenters back through the initial site of block at the scar interface.

Simulation 2: Panel D (1980ms) shows the wavefront 130ms following the final S1 beat. Here, the wavefront initially activates the more excitable healthy (sub-endocardial) myocardium, and slowly spreads and uniformly activates the fibrotic area extending through the midwall and towards the epicardium. Panel D (2530ms) shows the wavefront 80ms after the final S4 of the stimulus train. A strong heterogeneity in repolarisation is witnessed throughout the scarred region (following the S3 beat at 2100ms) as well as between the scar and the surrounding healthy myocardium, causing very slow, tortuous propagation through the scar and a zone of complete conduction block. Panel D (2900ms) then shows that as the healthy myocardium fully recovers from successful activation by the S4 beat it, may be re-activated by the wavefront that is still slowly 'hiding-out' in the scar.

References

[1] Maceira AM, et al. (2006). Normalized left ventricular systolic and diastolic function by steady state free precession cardiovascular magnetic resonance. *J Cardiovasc Magn Reson* 8:417-426.

[2] Halliday B, Baksi AJ, Galati A et al. (2018). Outcome in Dilated Cardiomyopathy Related to the Extent, Location and Pattern of Late Gadolinium Enhancement. J*ACC: Cardiovasc Imag*, 1–11.

[3] Muthalaly RG, Kwong RY, John RM et al. (2019) Left ventricular entropy is a novel predictor of arrhythmic events in patients with dilated cardiomyopathy receiving defibrillators for primary prevention. *JACC: Cardiovasc Imag*. 7:1177-1184

[4] Gould J, Porter B, Claridge S et al (2019) Mean entropy predicts implantable cardioverter-defibrillator therapy using cardiac magnetic resonance texture analysis of scar heterogeneity. *Heart Rhythm*, S1547-5271

[5] Fong C, Hazlett C, Imai K. Covariate balancing propensity score for a continuous treatment: Application to the efficacy of political advertisements. The Annals of Applied Statistics. 2018;12(1):156-77.

[6] Balaban, G., Halliday, B. P., Bai, W., Porter, B., Malvuccio, C., Lamata, P., et al. (2019). Scar shape analysis and simulated electrical instabilities in a non-ischemic dilated cardiomyopathy patient cohort. *PLoS Computational Biology*, *15*(10), e1007421–e1007421.

[7] TenTusscher K, Panfilov AV. (2006) Alternans and spiral breakup in a human ventricular tissue model. *Am J Physiol: Heart Circ*, 291:H1088–H1100.

[8] Vigmond EJ, Hughes M, Plank G et al (2003) Computational tools for modeling electrical activity in cardiac tissue. *J Electrocardiology*, 36:69–74.

[9] Bayer JD, Blake RC, Plank G et al. (2012) A novel rule-based algorithm for assigning myocardial fiber orientation to computational heart models. *Ann Biomed Eng*, 40:2243–2254.

[10] Anderson KP, Walker R, Urie R et al (1993) Myocardial electrical propagation in patients with idiopathic dilated cardiomyopathy. *J Clin Invest*, 92:122–140.

[11] Vigmond EJ, Pashaei A, Amraoui S, et al. (2016). Percolation as a mechanism to explain atrial fractionated electrograms and reentry in a fibrosis model based on imaging data. *Heart Rhythm*, 13:1536–1543.

[12] Glashan CA, Androulakis AFA, Tao Q et al. (2018) Whole human heart histology to validate electroanatomical voltage mapping in patients with non-ischaemic cardiomyopathy and ventricular tachycardia. *Euro Heart J*, 39:2867–2875.

[13] Betensky, B. P., Kapa, S., & Desjardins, B. (2013). Characterization of transseptal activation during septal pacing: criteria for identification of intramural VT substrate in nonischemic cardiomyopathy. *Circ Arrhyth Electrophys*, 6:1123-1130
